# Perinatal complications and schizophrenia: involvement of the immune system

**DOI:** 10.3389/fnins.2013.00110

**Published:** 2013-06-25

**Authors:** Trisha A. Jenkins

**Affiliations:** School of Medical Sciences, Health Innovations Research Institute, RMIT UniversityBundoora, VIC, Australia

**Keywords:** schizophrenia, animal models, cytokines, infection, hippocampus

## Abstract

The neurodevelopmental hypothesis of schizophrenia suggests that, at least in part, events occurring within the intrauterine or perinatal environment at critical times of brain development underlies emergence of the psychosis observed during adulthood, and brain pathologies that are hypothesized to be from birth. All potential risks stimulate activation of the immune system, and are suggested to act in parallel with an underlying genetic liability, such that an imperfect regulation of the genome mediates these prenatal or early postnatal environmental effects. Epidemiologically based animal models looking at environment and with genes have provided us with a wealth of knowledge in the understanding of the pathophysiology of schizophrenia, and give us the best possibility for interventions and treatments for schizophrenia.

## Introduction

Schizophrenia is a chronic, severely debilitating psychiatric disorder that affects ~1% of the population worldwide (Saha et al., 2005). A disease of early adulthood, schizophrenia is in general diagnosed between the ages of 15 and 30, with men developing the disorder slightly earlier than women (Sham et al., 1994; Hafner, 2003). Symptomology is characterized into several very different subtypes: positive symptoms, including hallucinations, delusions and paranoid thoughts (Andreasen et al., 1990); negative symptoms, such as avolition and anhedonia (Andreasen, 1982; Kirkpatrick et al., 2006); and cognitive deficits, that is poor executive function and attention, and impaired working memory (Heinrichs and Zakzanis, 1998).

The aetiology of schizophrenia is extremely complex and poorly understood but is believed to result from a combination of environmental and genetic factors (Brown, 2011a). The number of shared genes between a person with schizophrenia and a family member is strongly associated with a risk of developing schizophrenia, however, no degree of shared genes results in a certainty of developing the disorder (Mortensen et al., 2010). As such there is a growing body of evidence to suggest that environmental factors play a major role in the aetiology of schizophrenia, many of these being perinatal complications.

## The neurodevelopmental theory of schizophrenia

While schizophrenic symptoms manifest in young adulthood, the neurodevelopmental theory of schizophrenia suggests that an insult during neurodevelopment produces long-term detrimental effects on the brain. Findings from epidemiological studies provide evidence that perinatal factors including environment, parental influence on the foetus during pregnancy and obstetric complications contribute to the risk of developing schizophrenia.

### Time and place of birth

Two major epidemiological notions for a neurodevelopmental theory of schizophrenia are season of birth, and place of birth. Seasonal birth rates of schizophrenics differ from those of the general population with people born during winter or early spring, where there is a greater predominance of infections such as colds and influenza, having an increased risk of developing schizophrenia (Bradbury and Miller, 1985; Torrey et al., 1997).

Urbanicity, compared to being raised in a rural area, also gives rise to a greater risk of schizophrenia (Marcelis et al., 1999a; March et al., 2008) with the increase in susceptibility found before, rather than around, the time of illness onset (Marcelis et al., 1999b). Indeed individuals moving to a higher degree of urbanization during upbringing show an increased risk of schizophrenia (Pedersen and Mortensen, 2006), while moving rurally decreases the risk, suggesting that repeated exposures to candidate risk factors such as infection and exposure to toxins while either in the womb, or during development confer a greater risk toward psychosis (Pedersen and Mortensen, 2001). An alternative hypothesis is the influence of “cognitive social capital”; that is aspects of mutual trust, bonding and safety exert a developmental stress-related impact on the mental health of the children growing up in these urban environments (Krabbendam and van Os, 2005). Indeed the role of nutrition could also influence here. Both of these factors are discussed below.

### Prenatal exposure to infection

Research on prenatal infection and the aetiology of schizophrenia has been extensively reviewed by Brown (2006, 2011a, b), and Brown and Derkits (2010). Epidemiological and birth cohort studies show that infection is a credible environmental risk factor for schizophrenia with foetal exposure to viral or parasitic agents increasing one's susceptibility to the disorder.

Much of the evidence pertaining to within-womb infection and schizophrenia is from influenza studies. An initial study reporting an increased risk of schizophrenia in individuals that had been in the second trimester of development during the 1957 influenza pandemic (Mednick et al., 1988) was confirmed by others (O'Callaghan et al., 1991; Kunugi et al., 1995; McGrath and Castle, 1995), although a recent publication studies did not demonstrate an increased risk of schizophrenia among children exposed during any trimester or month of prenatal life of this epidemic (Selten et al., 2010). Alternatively, other influenza epidemics do support the maternal influenza hypothesis (Barr et al., 1990; Sham et al., 1992); with a further study, this time with birth cohort data, demonstrating that maternal influenza during the first half of pregnancy is found to be associated with an increase in susceptibility of the foetus to develop schizophrenia, in this case, a three-fold influence (Brown et al., 2004a).

Viral susceptibility is also demonstrated with maternal exposure to herpes simplex type 2 virus where elevated maternal IgG antibody to HSV-2 was measured in offspring who later developed psychotic disorders, including schizophrenia (Buka et al., 2001a). Further susceptibility exists with rubella, such that children born to mothers with clinical rubella were shown to have a 10- to 20-fold increased risk in developing schizophrenia (Brown et al., 2001). In addition, the parasite *Toxoplasma gondii* was associated with a 2 1/2-fold increase in risk of schizophrenia (Brown et al., 2005), confirmed by a later study (Mortensen et al., 2007b), and is also suggested as a risk factor for early-onset schizophrenia (Mortensen et al., 2007a).

### Maternal stress and nutrition influences

It is well known that maternal stress in pregnancy has long-term neurodevelopmental effects on the infant. The onset of schizophrenia has been associated with exposure of the pregnant mother to loss of the husband (Huttunen and Niskanen, 1978), undesired pregnancy (Myhrman et al., 1996), and threat and occurrence of war (Meijer, 1985; van Os and Selten, 1998). Elevated rates of schizophrenia are also related to maternal depression during pregnancy (Jones et al., 1998).

A role of prenatal malnutrition in schizophrenia has been demonstrated through ecological data collected from times of famine. Investigators of the Dutch Hunger Winter demonstrated a relationship between nutritional deprivation and schizophrenia (Susser et al., 1996). Further studies from China replicated these findings (St Clair et al., 2005; Xu et al., 2009). Obviously at times of famine there is also high stress so the implication that food scarcity is an absolute risk factor for schizophrenia should be treated with some caution. However, there is evidence that some micronutrient deficiencies including low homocysteine (Brown et al., 2007) and vitamin D (McGrath, 1999) increase the incidence of schizophrenia. When we consider these risks, a recognized consequence is growth retardation of the foetus. Low birth weight and smaller head circumference are indeed predictors of schizophrenia (Cannon et al., 2002a).

### Paternal age

Increasingly it is becoming evident that paternal age is a strong and significant predictor of schizophrenia diagnosis. Relative risk of schizophrenia reaches three times normal levels in offspring of men aged 50 years or more, independent of the mother's age (Malaspina et al., 2001). Conversely a significant increase in risk of schizophrenia in the offspring of younger fathers (less than 25 years of age) has been found, which could also be associated with an increased risk in males but not females (Miller et al., 2011).

### Obstetric complications

Complications of pregnancy and delivery show clear susceptibility for schizophrenia (Cannon et al., 2002a; Clarke et al., 2006), with individuals with schizophrenia more likely to have experienced hypoxia at birth (Geddes et al., 1999; Zornberg et al., 2000; Dalman et al., 2001). To add to this, foetal hypoxia is associated with greater structural brain abnormalities among schizophrenic patients, namely reduced gray matter and ventricular enlargement (Cannon et al., 2002b), compared to their non-schizophrenic siblings, with these anatomical anomalies possibly influenced, in part, by schizophrenia susceptibility genes (Van Erp et al., 2002).

## Gene-environment collaboration

While the environmental evidence pertaining to schizophrenia risk is strong, these environmental factors are deemed rarely sufficient to cause schizophrenia independently. It is suggested that they act in parallel with an underlying genetic liability, such that an imperfect regulation of the genome mediates these prenatal or early postnatal environmental effects (Maric and Svrakic, 2012). Researchers have identified a number of genetic variants that predispose the brain to developing schizophrenia, with vulnerability in DISC1 and NRG1 the best replicated in association with a developmental hypothesis.

Disrupted in schizophrenia 1 (DISC1) (Millar et al., 2000) is one of the most promising candidate genes for schizophrenia and other psychoses (Ishizuka et al., 2006). Many biological studies have indicated a role for DISC1 in early neurodevelopment and synaptic regulation, elegantly reviewed by Brandon and Sawa (2011). DISC1 regulates neuronal migration (Kamiya et al., 2005) and progenitor cell proliferation (Mao et al., 2009) in the developing cortex; and plays an important role in synapse formation and maintenance (Hayashi-Takagi et al., 2010). In a number of recent studies interactions between maternal infection and DISC1 have been demonstrated. In DISC1 genetic mice, maternal inflammation by Poly I:C caused deficits in object recognition and fear memories in adult offspring in DISC1 phenotype, but not wild type mice (Ibi et al., 2010; Nagai et al., 2011). These behavioral deficits were associated with decreased enlargement of ventricles, reduced volumes of the amygdala and periaqueductal gray matter, and decreased number of dendritic spines in the hippocampus (Abazyan et al., 2010); and a more pronounced release of IL-6, suggesting this may be important in the pathophysiology of this interaction (Lipina et al., 2013).

Neuregulin-1 (NRG1) is another candidate gene for schizophrenia (Stefansson et al., 2002). Expressed at central nervous system synapses, NRG-1 regulates various neurodevelopmental processes, including neuronal migration, myelination, synaptic plasticity, and neurotransmitter function (Mei and Xiong, 2008). A study investigating a population of carriers of the NRG1 (valine to leucine) mutation has observed increased circulating proinflammatory cytokines, including IL-6, TNF-α, and IL-1b suggesting a role of this mutation in immune dysregulation in schizophrenia (Marballi et al., 2010).

## Potential mechanisms

The suggested causes pertaining to the neurodevelopmental theory of schizophrenia are widespread. What is recognized is that all potential risks stimulate activation of the immune system.

### Cytokines

Cytokines are immunomodulatory proteins with many biological actions. Expression of cytokines in the brain points to multiple roles in physiological processes, including brain development (Deverman and Patterson, 2009). Cytokine alterations in schizophrenia have been comprehensively investigated in adult schizophrenic patients, with IL-1, 2, 6, 8, 10, and 12 all been observed to increase in schizophrenia, along with TNFα, TGF-β 1, and IFNγ (Gaughran, 2002; Drzyzga et al., 2006; Anderson et al., 2013).

When we consider perinatal influence, elevated plasma levels of IL-8 in mothers in their second half of pregnancy were shown to indicate an increased risk of schizophrenia in the foetus (Brown et al., 2004b). Moreover, maternal levels of TNF-α were significantly elevated among individuals with schizophrenia, with evidence of increasing odds of psychosis in relation to higher cytokine levels (Buka et al., 2001b). These researchers also demonstrated a significant association between increased levels of maternal serum IgG and IgM class immunoglobulins, both generated in response to infection, at delivery and the subsequent development of psychosis in offspring (Buka et al., 2001a).

### Microglia

The action of proinflammatory cytokines are medicated by microglia. Microglia, resident immune defenders of the central nervous system play important roles in the development and protection of neural cells. However, they can contribute to injury under pathological conditions as activated (Sternberg, 2006). While there is no direct evidence for microglia changes as a risk factor for schizophrenia, there are some reports of abnormal activation of microglia in post-mortem studies of schizophrenic brains (Bayer et al., 1999; Radewicz et al., 2000) which was localized to the hippocampus in an imaging study using a marker of activated microglia cells (Doorduin et al., 2009).

### Kynurenine

Activated microglia release a large number of inflammatory mediators thus sustaining neuroinflammation and neurotoxicity. The kynurenine pathway, the main pathway for tryptophan metabolism, is one of the major regulators of the immune response and may also be implicated in the inflammatory response in schizophrenia (Erhardt et al., 2007). Elevated levels of kynurenine have been reported in prefrontal cortex and cerebrospinal fluid of schizophrenic patients (Erhardt et al., 2001; Schwarcz et al., 2001; Nilsson et al., 2005), though at this stage no measures exist for those at-risk individuals.

In rodent studies, administration of kynurenine to mothers (GD15 to PD21) produced, in the offspring, reduced hippocampal glutamate levels and impaired avoidance spatial memory and attentional set-shifting, suggesting that increases in brain kynurenic acid during a vulnerable period in brain development may play a significant role in the pathophysiology of schizophrenia (Pocivavsek et al., 2012; Alexander et al., 2013). In a model of social isolation rearing, a neurodevelopmental animal model of schizophrenia (Fone and Porkess, 2008), plasma tryptophan and kynurenine are significantly elevated, while kynurenic acid is significantly decreased, suggesting a decrease in the neuroprotective, and an increase in the neurodegenerative, directed components of the pathway (Moller et al., 2012).

## Immune stimulation to brain pathology

The effects of these risk factors suggest that a variety of inflammatory mechanisms disrupt the expected normal development of the brain (depending on the insult this is of course at different time periods, and thus different neuronal populations could be affected); the consequence being a cascade of events producing behavioral abnormalities in the patient that lay dormant until long after the insult. As such there is no direct evidence of a foetal or perinatal brain tissue injury, but indirect pathology at both an anatomical and neurochemical level.

### Anatomy

A consistent finding in schizophrenia is dilation of the lateral ventricles (Wright et al., 2000), which is observed from onset of the disorder (Fannon et al., 2000). Moreover, patients have significantly less whole brain volume (Andreasen et al., 1994), again observed at the first evidence of symptomatology (Steen et al., 2006) which can be attributed to a reduction in gray matter (Rasser et al., 2010). Gross brain abnormalities have been identified consistently since the onset of imaging technology, with deficits observed in regions including medial temporal lobe structures, such as the amygdala, hippocampus, and parahippocampal gyrus, and neocortical temporal lobe regions; prefrontal cortex, and indeed the whole frontal lobe; as well as parietal lobe, cerebellum, thalamus, basal ganglia and corpus callosum [for review see Shenton et al. (2001); Shepherd et al. (2012)]. With such diffuse pathology it is proposed that connectivity reductions exist across the brain, which is suggestive of a global connectivity deficit in the brains of schizophrenic patients (Fornito et al., 2012).

Research on individuals at high risk to develop schizophrenia have demonstrated anatomically to have also shown gray matter deficits with the volume or cortical thickness of frontal cortex, temporal cortex, and amygdala–hippocampal limbic system regions altered (Jung et al., 2010). In addition, white matter, in terms of fiber integrity, is aberrant in these individuals (Karlsgodt et al., 2009; Bloemen et al., 2010).

At a cellular level, disruptions in neuronal cytoarchitecture have been observed in the brains of schizophrenia patients. Within the hippocampus, pyramidal neuron loss (Falkai and Bogerts, 1986; Jeste and Lohr, 1989) and pyramidal neuron disarray (Kovelman and Scheibel, 1984; Arnold, 2000) are observed, suggesting a dysfunction of neuronal migration in the embryonic period; while in the prefrontal cortex an increased neuronal density is observed in dorsolateral prefrontal cortex (Selemon et al., 1995), perhaps attributable to the trend for cortical gray matter to be thinner in schizophrenic brains (Kumari et al., 2008) and thus the neurons more densely packed. The principal subcortical input to dorsolateral prefrontal cortex is the mediodorsal nucleus of the thalamus, and reductions in neuron numbers in this region is also observed in schizophrenia (Popken et al., 2000).

Ultrastructural changes in neurons in schizophrenia have also been identified with decreased neuron size in limbic and temporal regions (Benes et al., 1991; Zaidel et al., 1997); abnormal dendritic spine densities in the cortex (Garey et al., 1998; Glantz and Lewis, 2000); and altered cortical cytoarchitecture (Arnold et al., 1991), perhaps in part due to abnormal synaptic pruning (Feinberg, 1982). Molecular changes related to synaptic function are consistently reduced with reports of decreased levels of pre-synaptic proteins such as synaptophysin and growth-associated protein-43 in both the prefrontal cortex and hippocampus (Glantz and Lewis, 1997; Eastwood and Harrison, 1998).

## Prodromal observations

Substantial evidence has demonstrated that at least a proportion of schizophrenia patients displayed subtle neuromotor, intellectual, social, or behavioral abnormalities well before the onset of psychosis. A higher rate of neuromuscular abnormalities, including less advanced motor skills, and involuntary movements and posturing of the upper limbs have been observed in pre-schizophrenia children when compared to their healthy siblings and age-matched children (Jones et al., 1994; Walker et al., 1994; Schiffman et al., 2004). Delayed language development is also observed in children at risk with more speech problems reported in the developing adolescent than non-at-risk peers (Jones et al., 1994). Moreover, the literature suggests that individuals who develop schizophrenia present poorer cognitive abilities in childhood than those who never develop schizophrenia. During childhood, individuals who subsequently developed schizophrenia, were reported to have a lower IQ (Woodberry et al., 2008) and worse academic performance (Jones et al., 1994; Fuller et al., 2002). Indeed with respect to school adjustment, children at risk of psychosis had significantly poorer adjustment than comparison adolescents (Dworkin et al., 1994; Shapiro et al., 2006) suggesting that a poor start to education may be in part a cause.

Impairments in social development in pre-schizophrenic children and adolescents have been noted in a variety of studies. Features of social withdrawal, anxiety and anhedonia in those individuals with elevated schizophrenia-spectrum personality disorder is often observed (Gruzelier and Kaiser, 1996; Blanchard et al., 2011). At risk and first episode patients significantly differ from normal subjects on measures of social functioning in the domains of peer, family, work, and school relationships (Ballon et al., 2007). From school age, poorer overall social competence are reported including poorer peer relationships (Dworkin et al., 1994), which may be attributed to immaturity and unpopularity with peers (Hans et al., 2000). Indeed, odd speech and impulsivity that was associated with late maturation was observed in female at-risk individuals (Gruzelier and Kaiser, 1996), as well as a decreased interest in extracurricular activities such as hobbies (Dworkin et al., 1994).

## Maternal infection animal models of schizophrenia

To explore the mechanisms by which early activation of the immune system might foster schizophrenia, researchers have turned to animal models in which the mother's immune system is activated by exposure to infectious agents that trigger the same kind of immune response as a bacterial or viral infection does and the brain and behavioral consequences of the prenatal immunological manipulation are then compared in the resulting offspring relative to offspring born to vehicle-treated control mothers (Patterson, 2009; Boksa, 2010; Meyer and Feldon, 2010).

### LPS

Systemic administration of the bacterial cell wall component from Gram negative bacteria lipopolysaccharide (LPS) during pregnancy in the rodent is a widely accepted model of bacterial infection, which activates the immune response including cytokine production, inflammation, and fever (Boksa, 2010). In behavior pertaining to schizophrenia, this model exhibits impairments in prepulse inhibition (Borrell et al., 2002) and working memory (Coyle et al., 2009), along with an increase in amphetamine-stimulated locomotor activity (Fortier et al., 2004a). Morphologically, LPS offspring demonstrate prefrontal and hippocampal pathology with reduced dendritic numbers, arbors and length, and abnormal excitation (Borrell et al., 2002; Lowe et al., 2008; Baharnoori et al., 2009; Cui et al., 2009). These studies have extended to administering LPS at early times of brain development. LPS administered on postnatal day 7 and 9 impaired object recognition memory in adulthood, and reduced expression of parvalbumin-immunoreactive GABAergic neurons in the hippocampus (Jenkins et al., 2009).

### Poly(I:C)

The inflammatory agent poly(I:C) (polyriboinosinic-polyribocytidilic acid), a synthetic analog of double-stranded RNA, stimulates the production and release of many pro-inflammatory cytokines (Fortier et al., 2004b). In the rodent prenatal poly(I:C) model, pregnant dams are exposed to polyI:C, with the resulting offspring displaying a multitude of behavioral, cognitive and pharmacological dysfunctions, dependant on the time of exposure (Meyer and Feldon, 2010). In brief, impairments in prepulse and latent inhibition, working memory and social behavior are observed, along with hyperactivity and psychostimulant sensitization in the offspring of polyI:C infected mothers (Shi et al., 2003; Zuckerman et al., 2003; Ozawa et al., 2006; Meyer et al., 2008b; Wolff and Bilkey, 2008). Concurrent neuronal deficits are also detected with reduced Purkinje cell density in the cerebellum (Shi et al., 2009), delayed myelination in the hippocampus (Makinodan et al., 2008), depleted corticoneurogenesis (Bitanihirwe et al., 2010; Feldon et al., 2010) and a reduction in GABAergic, glutamatergic, serotonergic, and dopaminergic markers (Nyffeler et al., 2006; Ozawa et al., 2006; Meyer et al., 2008a; Winter et al., 2009).

### Poly(I:C) and DISC1

A study that begins to grapple with the gene-environment collaboration of the neurodevelopmental theory of schizophrenia used treatment of poly(I:C) at gestation day 9, middle/end of the first trimester of human pregnancy, to mhDISC1 (mutant human disrupted-in-schizophrenia 1) mice. Researchers demonstrated that prenatal interaction of immune activation with mhDISC1 produced elevated anxiety, increased immobility in the forced swim test, and abnormal social behavior along with decreased corticosterone levels, suggesting an impaired stress response. In the hippocampus, treatment with poly I:C significantly increased levels of serotonin in the hippocampus of mhDISC1 mice, while decreasing serotonin turnover and reducing spine density on the dendrites of granule cells of the dentate gyrus (Abazyan et al., 2010).

### Prenatal stress

As discussed earlier, maternal stress in pregnancy increases the risk for onset of schizophrenia. Several groups have shown alteration in the neuroinflammatory response in rodent models of prenatal stress. Pups from restrained pregnant rat mothers at adulthood show increased activity of the cellular immune system, demonstrated by increased levels of CD8+ T cells and natural killer cells in the blood, and an exacerbated release of interferon-γ after mitogen stimulation (Vanbesien-Mailliot et al., 2007). In further studies with restrained pregnant mice mothers, offspring showed increased interleukin 1β and tumor necrosis factor-α levels in the hippocampus, increased interleukin 1β -immunoreactive microglial cells and increased activated microglia. In addition, systemic administration of LPS induced a significant increase in levels of inflammatory markers in the hippocampus of prenatally stressed mice but not of non-stressed animals (Diz-Chaves et al., 2012, 2013).

### Role of antipsychotics

Antipsychotic drugs are used to treat psychosis in schizophrenia but they have also been connected with normalization of some immune parameters in some animal models of schizophrenia. For example, chronic administration of chlorpromazine or clozapine reduced the attentional deficit observed in prenatal LPS-treated rats, while also normalizing enhanced levels of IL-1b, IL-2, and TNF-α in the offspring of LPS-treated mothers (Basta-Kaim et al., 2012). In other studies, aripiprazole ameliorated the impact of prenatal immune activation on offspring of poly I:C-treated dams in the amphetamine-sensitization locomotor model (Richtand et al., 2012), while paliperidone and risperidone normalized prefrontal cortical basal extracellular glutamate in the offspring of poly I:C treated mothers (Roenker et al., 2011).

## Conclusions

Epidemiological studies have demonstrated that prenatal exposure to infection or some kind of early traumatic event plays a role in the aetiology of schizophrenia. As these perinatal events are broad and their effect not comprehensive, the role of genetics must be considered—a gene-environment interaction; or perhaps particular genes producing a predisposition to an augmented effect of an environmental exposure. Epidemiology-based animal models investigating maternal exposure have given important insights in relation to the aetiology and pathophysiology of schizophrenia. Further animal studies linking candidate genes with environmental exposures will be indispensable in testing the hypothesis of causality in human epidemiological associations of gene and environment.

Further, what is clear is that while schizophrenic symptoms manifest in young adulthood, there is prodromal intellectual and behavioral abnormalities present in at least a population of susceptible children which we believe is due to indirect pathology at both an anatomical and neurochemical level that is evident from early childhood. Using animal models to understand what impact the environment has on brain pathology and behavior as the animal ages may elucidate the on-going debate of what is happening when.

We also must accept that schizophrenia is a disorder with different manifestations of the symptomatology in different patients, perhaps suggesting that differentiating types of schizophrenia based on clinical symptoms will help to determine different causes. From the elegant animal studies by Meyer and colleagues we know that the timing of the prenatal/perinatal environmental manipulation induces different phenotypes in neurodevelopmental animal models. As such particular environmental factors may have differential effects on neurodevelopmental processes depending on the stage of brain development, thus leading to different behavioral pathologies in adulthood.

The use of epidemiological-based animal neurodevelopmental models that reflect actual environmental insults offer the greatest promise in answering the what, why, and when questions of schizophrenia.

### Conflict of interest statement

The author declares that the research was conducted in the absence of any commercial or financial relationships that could be construed as a potential conflict of interest.
